# Pharmacogenomics-based systematic review of coronary artery disease based on personalized medicine procedure

**DOI:** 10.1016/j.heliyon.2024.e28983

**Published:** 2024-03-29

**Authors:** Siamak Kazemi Asl, Milad Rahimzadegan, Alireza Kazemi Asl

**Affiliations:** a Deputy of Education, Ministry of Health and Medical Education, Tehran, Iran; b Functional Neurosurgery Research Center, Shohada Tajrish Comprehensive Neurosurgical Center of Excellence, Shahid Beheshti University of Medical Sciences, Tehran, Iran

**Keywords:** Coronary artery disease, Pharmacogenetics, Drug, GWAS, Variant

## Abstract

**Background:**

Coronary artery disease (CAD) is the most common reason for mortality and disability-adjusted life years (DALYs) lost globally. This study aimed to suggest a new gene list for the treatment of CAD by a systematic review of bioinformatics analyses of pharmacogenomics impacts of potential genes and variants.

**Methods:**

PubMed search was filtered by the title including Coronary Artery Disease during 2020–2023. To find the genes with pharmacogenetic impact on the CAD, additional filtrations were considered according to the variant annotations. Protein-Protein Interactions (PPIs), Gene-miRNA Interactions (GMIs), Protein-Drug Interactions (PDIs), and variant annotation assessments (VAAs) performed by STRING-MODEL (ver. 12), Cytoscape (ver. 3.10), miRTargetLink.2., NetworkAnalyst (ver 0.3.0), and PharmGKB.

**Results:**

Results revealed 5618 publications, 1290 papers were qualified, and finally, 650 papers were included. 4608 protein-coding genes were extracted, among them, 1432 unique genes were distinguished and 530 evidence-based repeated genes remained. 71 genes showed a pharmacogenetics-related variant annotation in at least (entirely 6331 annotations). Variant annotation assessment (VAA) showed 532 potential variants for the final report, and finally, the concluding PGs list represented 175 variants. Based on the function and MAF, 57 nonsynonymous variants of 29 Pharmacogenomics-related genes were associated with CAD.

**Conclusion:**

Conclusively, evaluating circulating miR33a in individuals’ plasma with CAD, and genotyping of rs2230806, rs2230808, rs2487032, rs12003906, rs2472507, rs2515629, and rs4149297 (ABCA1 variants) lead to precisely prescribing of well-known drugs. Also, the findings of this review can be used in both whole-genome sequencing (WGS) and whole-exome sequencing (WES) analysis in the prognosis and diagnosis of CAD.

## Highlights

• Coronary artery disease (CAD) is the most common cause of disability-adjusted life years (DALYs).
• Genetic biomarkers are increasingly introduced for the diagnosis and treatment of CAD.
• This review mainly focuses on recent reports of genomics and proteomics data associated with CAD.
• This review covers the recent pharmacogenomics-associated genes, variants, and miRNAs with CAD.
• Potential variants for personalized medicine and miRNAs for detection of CAD are discussed.

## 1 Introduction

Coronary artery disease (CAD) is the foremost reason of death and loss of Disability Adjusted Life Years (DALYs) worldwide. Low and middle-income countries bear a disproportionate share of this burden, accounting for almost 7 million deaths and 129 million DALYs each year [[1], [2], [3], [4], [5]]. CAD was responsible for 8.9 million fatalities and 164.0 million DALYs in 2015 [6]. Survivors of Myocardial Infarction (MI) are at increased risk of repeated infarction and have a five to six times higher yearly death rate than non-CAD patients [[7], [8], [9], [10], [11]]. Overall, the age-adjusted prevalence of MI hospitalization was 215/100,000 people between 1979 and 1981, then grew until 1987, stable in the next decade, and then began to fall beginning in 1996, reaching 242/100,000 people in 2005 [7,[12], [13], [14], [15]]. Despite the fact that the incidence of death from CAD has dropped over the previous four decades, it still accounts for over one-third of fatalities in those over the age of 35. Nearly fifty percent of the decrease in mortality can be attributed to improved therapy of the acute phase of ACS and accompanying comorbidities such as acute heart failure, enhanced primary and secondary preventive methods, and chronic angina revascularization [7]. Different geographical locations might possess a genetic susceptibility to CAD risk factors, like metabolic syndrome, which is a risk factor in South Asia [16,17].

Significant progress has been achieved in understanding the genetics of cardiovascular disease (CVD) during the previous decade. McPherson defined that the genetic architecture of CAD is mostly driven by the combined impact of numerous common variants, each of which contributes little to disease risk when considered separately. This is in contrast to rare variants having significant influences on the incidence of coronary disease [18]. The identification of these common variations follows the publication of massive genome-wide association studies (GWAS) on a bigger view [19,20]. Since 2007, genome-wide association studies (GWAS) have found links between CAD and 321 chromosomal loci [21,22]. By examining the whole genome, CAD GWASs discovered numerous novel genes with earlier unrecognized importance for atherosclerosis and retrieved existing therapy candidates and genes recognized to raise disease risk. These findings suggest new disease-causing pathways. The extensive outcomes of GWAS research are increasingly being examined for translational reasons in the post-GWAS age. The goals of these investigations are (a) understanding the disease-related processes underlying CAD loci; (b) prioritizing causative genes and prospective innovative therapeutic targets; and (c) using CAD genetic variants for stratification incidence, prevention of disease, and personalized medicine [23]. GWAS is designed to have the greatest sensitivity for finding impacts of common single-nucleotide polymorphisms (SNP). Simultaneously growing sequence coverage of the human genome permitted the detection of several variants with low frequencies, allowing researchers to investigate whether the signals at risk loci are caused by common or uncommon variants. The findings fascinatingly and unmistakably showed that the common variants with small impact sizes are mostly responsible for the overall genetic predisposition to CAD identified through GWAS sites [24]. These common variations are often found in assumed regulatory regions of the genome, which means they usually alter gene expression and hence the complicated network which links the function of numerous genes [23]. Further research, such as whole-exome sequencing (WES) and exome array, focused on the gene encoding sequences. Rare loss-of-function mutations in ANGPTL4, LPL, and SVEP1 that are connected to CAD were found by Stitziel et al. pointing to potential new treatment targets [25]. While ANGPTL4 and LPL are already being explored in the management of hypertriglyceridemia, SVEP1, a not well-known gene, has been functionally verified to have an atheroprotective impact on mice [[26], [27], [28]]. WES was employed in a study to discover rare variants for CAD/MI in a noticeable patient cohort of 9793 individuals [29]. Particularly rare APOA5 and LDLR alleles demonstrated an exome-wide significant correlation with MI (p 8107), indicating that a larger sample size may be necessary to identify rarer variants. Unite Kingdom BioBank (UKBB) will provide ES data from 200,000 people by the end of 2020. More unique uncommon variations for CAD are likely to be uncovered using comparable datasets from different biobanks, which will help in treatment discovery [23].

Abundant CAD-related genes detected by GWAS may be druggable, as evidenced by the fact that many candidate responsible genes at CAD loci are medical drug targets, including 3-hydroxy-3- methylglutaryl-coenzyme A reductase (HMGCR) (statins), APOB (Mipomersen), and PCSK9 (respective antibodies or inhibitors), as well as gene targets currently undergoing pre-clinical evaluations [30,31]. Indeed, associations between rare polymorphisms and CAD risk, GWAS findings, and associated system genetics are progressively being exploited for preclinical target prioritization and medication selection [29,[32], [33], [34]]**.** Loss-of-function mutations in ANGPTL4 (angiopoietin-like 4), a locally produced LPL inhibitor, have been correlated with hypolipidemia and atheroprotection. Several ANGPTL4 inhibitors have recently been in clinical studies and have indicated promise in reducing atherogenic dyslipidemia. As well as lipid metabolism genes, efforts have been made to investigate potential targets connected to GWAS results impacting inflammation and arterial wall dynamics [35]. Tragante et al. used accessible GWAS data on CAD to find drug-gene interactions and ranked options from a pool of current treatments (drug repurposing) [32]. According to the DrugBank (https://go.drugbank.com/), there are 21 genes with complete status of clinical trials for an FDA-approved drug related to CAD treatment which emphasizes the Pharmacogenetic importance of clinical and experimental findings. Based on these clues, and the lack of a comprehensive and concentrated study in pharmacogenomics of CAD in personalized medicine strategy of treatment, the present study systematically reviewed the recent reports of literature and carried out a new in silico analysis suggesting a potential druggable variant list for further studies and designed a gene panel of PGx-CAD for WES analysis.

## 2 Methods

### 2.1 Data collection and preparation

PubMed search [November 22nd, 2023] was filtered by the title including Coronary Artery Disease from 2020 to 2023 and 5618 articles were found. In the next step, based on the inclusion criteria and exclusion criteria second-layered filtration was carried out. Inclusion criteria were meta-analyses, review articles, original articles, GWAS, pharmacogenomics, and personalized medicine reports. The exclusion criteria were as follows: case reports, involving another disease such as opioid addiction, alcohol dependence, type 1 and type 2 diabetes, stroke, hyperthyroidism, hypertension, hyperuricemia, kidney disease, renal dysfunction, COVID-19, metabolic baseline, microbial infections, animal models (mouse, rat, etc.), menopause status, pneumonia, bowel, dietary pattern, Familial hypercholesterolemia, stress, anxiety, cancer, Kawasaki disease, and non-biological items including the association of walking, food, and internet with CAD.

### 2.2 Bioinformatics analyses

The current review aimed to uncover potential interactions and novel results by combining the raw data extracted from the recent Pubmed publications and analyzing the data in a superior level of in silico predictions. All of the in silico analyses were carried out by STRING-MODEL ver. 12 (https://string-db.org/), Cytoscape ver. 3.10 (https://cytoscape.org/), miRTargetLink.2. (https://ccb-compute.cs.uni-saarland.de/mirtargetlink2), and NetworkAnalyst (https://www.networkanalyst.ca/NetworkAnalyst/). Also, for the final filtration step, PharmGKB (https://www.pharmgkb.org/) was utilized to exclude the genes with no pharmacogenetic effect based on the documented significant variant annotations.

## 3 Results

### 3.1 Data preparation

**Fig. 1:**
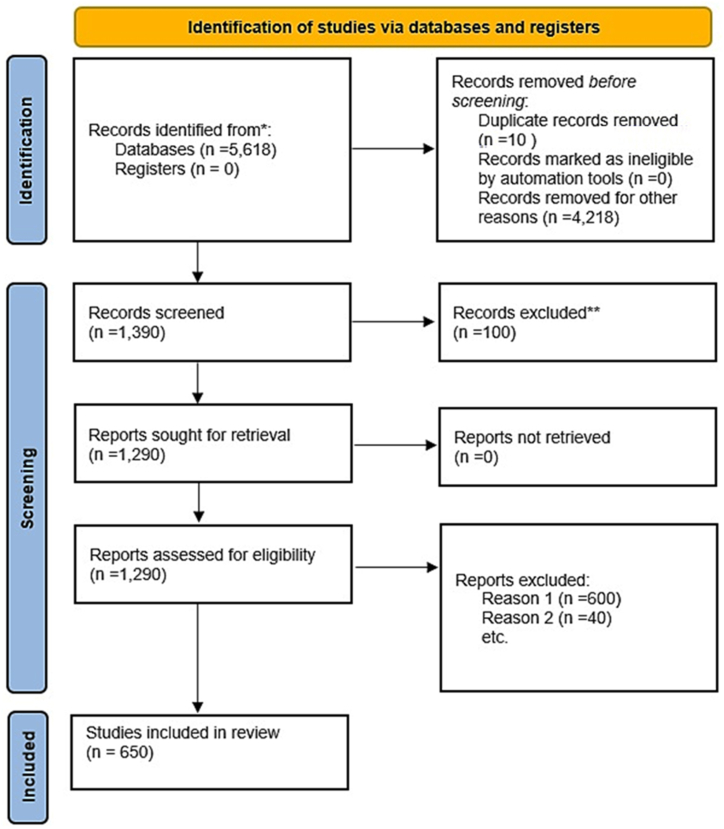
PRISMA flowchart of the systematic review for coronary artery disease from 2020 to 2023 publications in PubMed database. ** the excluded records were the papers with no direct focus on Coronary Artery Disease (CAD); and the Reason 1 for exclusion was the involvement of other disease such as opioid addiction, alcohol dependence, type 1 and type 2 diabetes, stroke, hyperthyroidism, hypertension, hyperuricemia, kidney disease, renal dysfunction, COVID-19, metabolic baseline, microbial infections; finally, the Reason 2 was case reports and animal models.

After deep investigation, 1290 papers were eligible for further insights, and 647 articles were discarded based on the exclusion criteria mentioned in the methods section. Also, 650 papers were included in the current review (Fig. 1). In total, 4608 protein-coding genes were found in 650 included papers. Many duplications were found; from which the most repeated genes were CRP (181 times), IL6 (129 times), TNF (99 times), ACE (97 times), APOB (89 times), IL1B (73 times), INS (70 times), APOE (64 times), APOA1 (59 times), and PCSK9 (52 times). By removing the duplications, 1432 unique genes were remained. Further analyses were performed on these final genes. To reach the genes which have pharmacogenetic impacts on the CAD, additional filtrations were considered according to the variant annotations. In the following sections, Protein-Protein Interactions (PPIs), Gene-miRNA Interactions (GMIs), Protein-Drug Interactions (PDIs), and variant annotation assessments (VAAs) were performed with details.

### 3.2 Protein-Protein Interactions (PPIs)

**Fig. 2:**
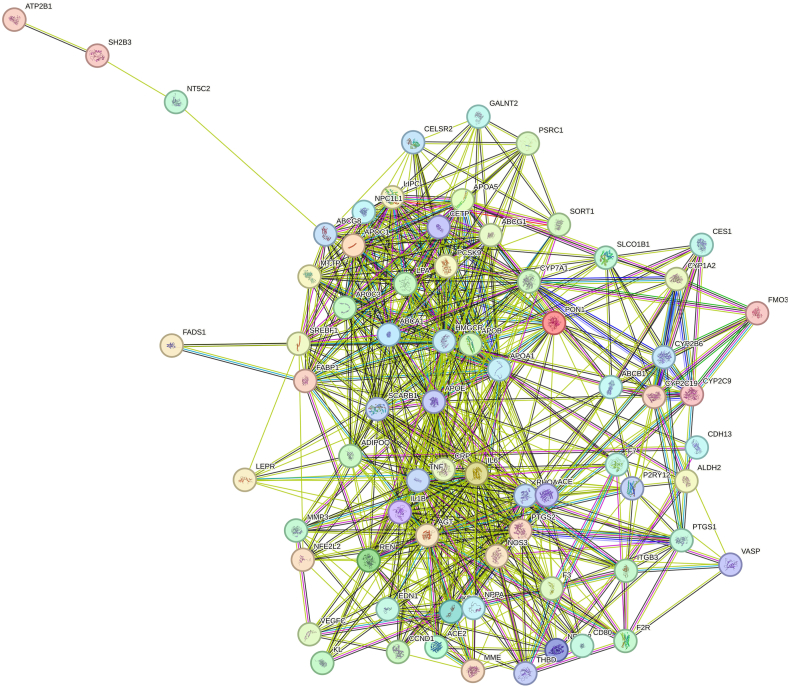
STRING-MODEL of 71 genes with pharmacogenomics impacts in coronary artery disease risk.

**Fig. 3:**
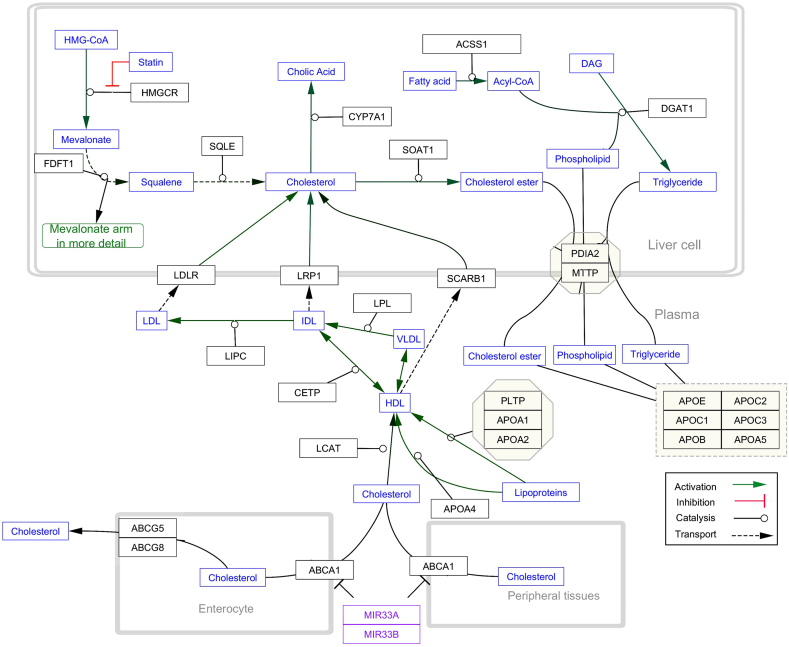
The most significant signaling pathway of 71 genes resulted from Cytoscape.

Primarily, STRING-MODEL was performed for 1432 proteins to find the unrelated and related proteins. Some proteins did not interact in the superior network including C2CD4C, EMP2, HyLS1, ATPSCKMT, and TEKT5. PPI enrichment p-value was <1.0e-16. To be more reliable and focused, only replicated proteins (genes) were selected for further steps of analysis. Thus, 898 unreplicated genes were eliminated and 530 evidence-based repeated genes remained. STRING-MODEL showed a completely interacted network of these 530 proteins (Figure not shown). The results of Cytoscape also represented the most significant curated pathways including malignant pleural mesothelioma (p-value: 2.45e-35), statin inhibition of cholesterol production (p-value:1.2e-15), and platelet-mediated interactions with vascular and circulating cells (3.34e-6). To reach a targeted gene list, the next step of filtering was finding the genes with pharmacogenomics effects which was possible through searching the related variant annotations with blood situation in the PharmGKB (https://www.pharmgkb.org/). Searching for genes with pharmacogenetic annotations represented that among 530 genes, 71 genes had a pharmacogenetics variant annotation at least. STRING-MODEL output of these PGx confirmed the fully-interacted proteins (Fig. 2). Cytoscape analysis displayed that statin inhibition of cholesterol production had the highest score compared with the other pathways (p-value = 2.87e-20) (Fig. 3). This pathway indicates noticeable genes and miRNAs including *ABCA1, APOA4, APOB, APOC1, APOC2, APOC3, APOC5, ABCG1, ABCG5, CETP, SCARB1, CYP7A1,* hsa-miR-33a, and hsa-miR-33b.

### 3.3 Gene-miRNA interactions (GMI)

**Fig. 4:**
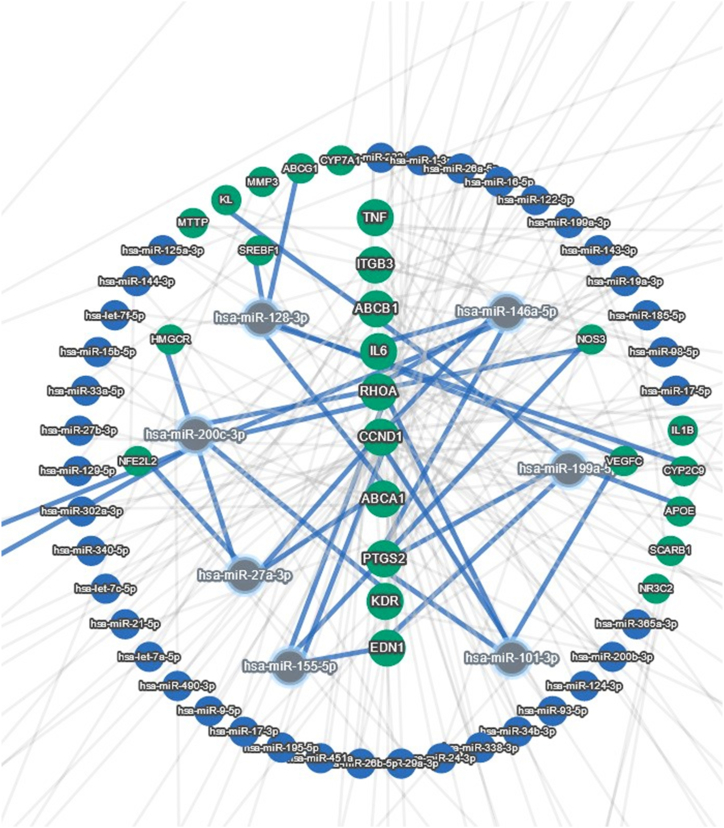
Concentric model of gene-miR interactions (GMI) visualized by miRTargetLink2.

The final 71 pharmacogenomics-based genes were then tested by the miRTargetLink2 to uncover the novel plausible gene-miR interactions. The concentric model was adjusted for only the strong evidence-based interactions and the central genes in this model were *TNF, ITGB3, ABCB1, IL6, RHOA, CCND1, ABCA1, PTGS2, KDR*, and *EDN1*. Notably, hsa-miR-146a-5p, has-miR128–3p, has-miR-101–3p, has-miR27a-3p, has-miR155–5p, has-miR199a-5p, and has-miR200c-3p had the most interacted miRNAs in this GMI network (Fig. 4). Interestingly, hsa-miR-33a was common in Fig. 2, Fig. 3.

### 3.4 Protein-Drug interaction (PDI)

**Fig. 5:**
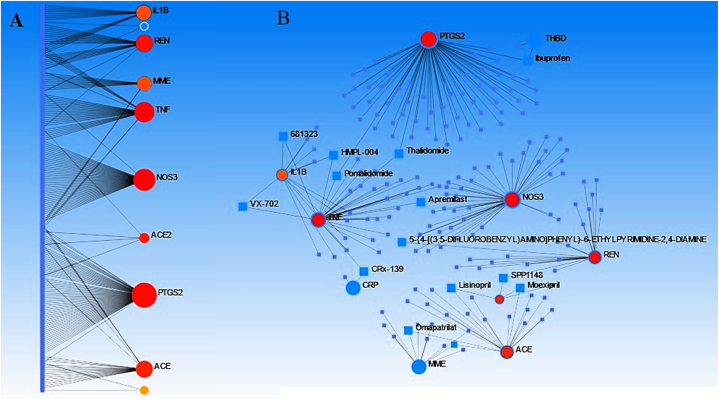
Protein-drug interactions (PDIs) of the genes with the most drug interactions illustrated by NetworkAnalyst server both in general (A) and detailed (B) networks.

Via NetworkAnalyst (https://www.networkanalyst.ca/NetworkAnalyst/), another in silico prediction was done for Protein-Drug interaction (PDI) among the 71 genes. The results of this bioinformatics data signified an interesting PDI among PTGS2, NOS3, TNF, REN, MME, IL1B, and ACE. According to this network, there are some linking drugs which could be considered as the potential candidate for CAD management such as Pomalidomide, Thalidomide, HMPL-004, 681,323, VX-702, Ampremilast, CRx-139, 5-6-ETHYLPYRIMIDINE-2,4-DIAMINE, SPP1148, Moexipril, Lisinopril, and Omapatrilat [1] (Fig. 5 A, B).

### 3.5 Variant annotations assessments (VAA)

To suggest the right gene list for pharmacogenes (PGs) associated with CAD, the current study examined the 6331 annotations of related variants of 71 PGs. The inclusion criteria for potentially related variants were as follows: the significant level specified with yes and also; the p-value should be lower than the 0.05 threshold. Findings from VAA indicated that 532 variants had the potential to be considered. Therefore, the final PGs list according to the deeply matched annotations with CAD showed 175 variants divided into three categories according to the dbSNP (https://www.ncbi.nlm.nih.gov/snp/) and Ensembl (http://www.ensembl.org/index.html). The first category consists of structural (missense and nonsynonymous) variants. Additionally, we searched for the clinical trial status of related drugs related to CAD. The completed status of clinical trials for each drug represents its reliability for prescribing based on the genotypes of individuals with CAD (Table 1). The second category consists of non-coding regulatory variants including Promoter, Transcription factor binding, enhancer, CTCF, and splicing (Table 2); and the third category comprises intronic, intergenic, upstream, and downstream variants which might not be described by molecular changes at least yet but may be in the future (Table 3). 57, 33, and 80 variants are indicated with genomic details in Table 1, Table 2, Table 3, respectively. The results summarized in the Tables can be utilized to highlight the remarkable actionable variants and consequently their genes. Both variant function and minor allele frequency (MAF) can prioritize some genes in the more potential PGx. In other words, the missense variants and variants with higher MAF (at least >0.05) can be listed in a group for exome gene panels with pharmacogenetic effects. According to Table 1 which indicates the missense or nonsynonymous variants, 29 genes can be prioritized including *ABCA1, ABCB1, ACE, AGT, ALDH2, APOB, APOC1, APOE, CES1, CYP2B6, CYP2C9, EDN1, F7, FABP1, FMO3, ITGB3, KDR, LEPR, NOS3, NPPA, NR3C2, NT5C2, P2RY12, PON1, PTGS1, SCARB1, SH2B3, SLCO1B1,* and *TLR4*. These genes can be considered for WES analysis for CAD patients or detecting CAD risk in healthy individuals as prognostic data.

**Table 1:** Variant annotation assessment including the structural variants of pharmacogenes-associated with CAD.

Genes	Variant	Function	MAF	Drugs	Clinical trial for CAD	Association	PMID
ABCA1	rs2230806	missense	0.44	Pravastatin	Completed	Genotype TT is associated with increased HDL-cholesterol when treated with pravastatin in people with Coronary Disease as compared to genotype CC.	19673941
ABCA1	rs2230808	missense	0.46	Fenofibrate	Unknown	Genotype CC is associated with increased response to fenofibrate in people with Hypertriglyceridemia as compared to genotype TT.	PMC3598593
ABCB1	rs2032582	missense	0.33	Clopidogrel	Completed	Allele T is associated with increased risk of Hemorrhage when treated with clopidogrel in people with Coronary Artery Disease as compared to allele A.	28589365
ABCB1	rs1045642	missense	0.4	Clopidogrel	Completed	Genotypes AA + AG are associated with decreased response to clopidogrel in people with Acute coronary syndrome as compared to genotype GG.	25542807
ABCB1	rs1128503	synonymous	0.42	Simvastatin	Completed	Genotype GG is associated with decreased response to simvastatin in people with Hypercholesterolemia as compared to genotypes AA + AG.	16321621
ABCG8	rs11887534	missense	0.06	–	–	Genotype CG is associated with increased risk of Coronary Artery Disease as compared to genotype GG.	PMC3833422
ACE	rs4343	synonymous	0.36	–	–	Allele G is associated with increased ACE activity in people with Hypertension as compared to allele A.	20066004
AGT	rs4762	missense	0.1	Benazepril	Unknown	Allele G is associated with increased response to benazepril in people with Hypertension.	17261659
AGT	rs699	missense	0.29	Irbesartan	Unknown	Genotype GG is associated with decreased likelihood of Acute coronary syndrome when exposed to Antiinflammatory agents, non-steroids in people with Acute coronary syndrome as compared to genotypes AA + AG.	20538124
ALDH2	rs671	missense	0.04	Nitroglycerin	Completed	Genotypes AA + AG is associated with decreased response to nitroglycerin in children with Heart Defects, Congenital as compared to genotype GG	31250045
APOB	rs676210	missense	0.37	Fenofibrate	Unknown	Genotype AA is associated with increased response to fenofibrate in people with Hypertriglyceridemia as compared to genotypes AG + GG.	PMC2952572
APOB	rs1801701	missense	0.04	Irbesartan	Unknown	Genotype CC is associated with increased response to irbesartan in people with Hypertrophy, Left Ventricular as compared to genotypes CT + TT.	15614026
APOB	rs1367117	missense/promoter	0.13	Irbesartan	Unknown	Genotype GG + AG is associated with response to irbesartan in people with Hypertension. Allele G is associated with increased risk of Hemorrhage when treated with warfarin in people with heart valve replacement.	PMC524175
APOB	rs13306198	stopgained	0.01	Apixaban; dabigatran; edoxaban; rivaroxaban	Unknwon; Terminated; Active Not Recruiting; Active Not Recruiting	Genotypes AA + AG is associated with increased likelihood of Hemorrhage when treated with apixaban, dabigatran, edoxaban or rivaroxaban as compared to genotype GG.	36145636
APOC1; APOE	rs429358	missense	0.15	Warfarin	Completed	Allele T is associated with LDL-C response when treated with atorvastatin in people with Coronary Disease. Genotypes CC + CT is associated with increased Hypertriglyceridemia in people with Coronary Disease or Hypertension as compared to genotype TT.	20031582
APOC1; APOE	rs7412	missense	0.08	Warfarin	Completed	Genotypes CT + TT is associated with increased percent reduction in LDL-cholesterol when treated with atorvastatin or pravastatin in people with Acute coronary syndrome as compared to genotype CC, etc.	19667110
CES1	rs146456965	missense	0.01	Clopidogrel; enalapril; sacubitril	Completed; Unknown; Unknown	Allele A is associated with decreased metabolism of when assayed with clopidogrel, enalapril and sacubitril in HEK cells as compared to allele C.	PMC5637814
CES1	rs202001817	missense	0.01	Clopidogrel	Completed	Allele A is associated with decreased enzyme activity of when assayed with clopidogrel in HEK cells as compared to allele G.	PMC5637814
CES1	rs202121317	missense	0.06	Enalapril	Unknown	Allele C is associated with decreased enzyme activity of when assayed with enalapril in HEK cells as compared to allele A.	PMC5637814
CES1	rs71647871	missense	0.03	Clopidogrel	Completed	Genotype CT is associated with decreased on-treatment ADP-induced platelet aggregation when treated with clopidogrel in people with Coronary Disease as compared to genotype CC, etc.	PMC3682407
CES1	rs201065375	missense	0.01	Clopidogrel; enalapril; sacubitril	Completed; Unknown; Unknown	Allele A is associated with decreased metabolism of clopidogrel, enalapril and sacubitril in HEK cells as compared to allele G.	PMC5637814
CES1	rs2307240	missense	0.05	Clopidogrel	Completed	Genotypes CT + TT are associated with increased response to clopidogrel in people with Acute coronary syndrome as compared to genotype CC.	PMC5543069
CES1	rs143718310	missense	0.01	Clopidogrel; enalapril; sacubitril	Completed; Unknown; Unknown	Allele G is associated with decreased metabolism of when assayed with clopidogrel, enalapril and sacubitril in HEK cells as compared to allele T.	PMC5637814
CES1	rs200707504	missense	0.01	Clopidogrel; enalapril; sacubitril	Completed; Unknown; Unknown	Allele C is associated with decreased enzyme activity of when assayed with clopidogrel, enalapril and sacubitril in HEK cells as compared to allele T.	PMC5637814
CES1	rs151291296	stopgained	0.01	Clopidogrel; enalapril; sacubitril	Completed; Unknown; Unknown	Allele C is associated with decreased enzyme activity of when assayed with clopidogrel, enalapril and sacubitril in HEK cells as compared to allele A.	PMC5637814
CETP	rs5882	missense	0.47	Simvastatin	Completed	Allele A is associated with increased response to simvastatin in people with Hypercholesterolemia as compared to allele G./Allele G is associated with increased response to rosuvastatin as compared to allele A.	17931083
CYP2B6	rs8192709	missense	0.05	Cyclophosphamide	Unknown	Genotypes CT + TT are associated with increased risk of hemorrhagic cystitis when treated with cyclophosphamide in people with recipients of HLA-identical hematopoietic stem cell transplantation as compared to genotype CC.	19005482
CYP2B6	rs3745274	missense	0.32	–		Genotype TT is associated with decreased expression of CYP2B6 as compared to genotypes GG + GT.	PMC4931886
CYP2C9	rs1057910	missense	0.05	–		Allele C is associated with decreased risk of Coronary Artery Disease as compared to allele A, etc.	21047199
EDN1	rs5370	missense/ctcf/promoter	0.25	Atenolol; irbesartan	Completed; Unknown	Genotypes GT + TT are not associated with response to atenolol and irbesartan in women with Essential hypertension as compared to genotype GG.	15188945
F7	rs6046	missense	0.14	Warfarin	Completed	Genotype AA is associated with increased sensitivity and risk of over-anticoagulation to warfarin during induction when treated with warfarin as compared to genotypes AG + GG.	22071881
FABP1	rs2241883	missense	0.22	Fenofibrate	Unknown	Genotypes CC + CT are associated with increased risk of Hypertriglyceridemia when treated with fenofibrate in people with Hypertriglyceridemia as compared to genotype TT, etc.	15249972
FMO3	rs1736557	missense/enhancer	0.1	Clopidogrel	Completed	Genotype AA is associated with decreased risk of high on-treatment platelet reactivity when treated with clopidogrel in people with Coronary Artery Disease as compared to genotypes AG + GG, etc.	33089397
ITGB3	rs5918	missense	0.09	Aspirin	Completed	Genotype CT is associated with increased blood loss when exposed to aspirin in people with Coronary Artery Disease as compared to genotype TT, etc.	16153930
KDR	rs1870377	missense	0.21	Sorafenib	Unknown	Allele T is associated with increased risk of Hypertension when treated with sorafenib as compared to genotype AA.	PMC2913951
LEPR	rs1805094	missense	0.14	Atorvastatin	Completed	Genotype GG is associated with increased bone marrow density in the lumbar spine when treated with atorvastatin in people with Acute coronary syndrome.	19023160
LEPR	rs1137101	missense	0.42	Simvastatin	Completed	Genotype GG is associated with increased percentage change in HDL-C levels when treated with simvastatin in people with Coronary Disease as compared to genotype AA.	18854995
LPA	rs3798220	missense	0.05	Aspirin	Completed	Genotype CT is associated with decreased risk of Myocardial Infarction when treated with aspirin in women.	PMC2678922
NOS3	rs1799983	missense	0.18	Aspirin; Beta Blocking Agents; clopidogrel; hmg coa reductase inhibitors	Completed; -, Completed,-	Allele T is associated with increased risk of in-stent restenosis when treated with aspirin, Beta Blocking Agents, clopidogrel and hmg coa reductase inhibitors in men with Coronary Artery Disease as compared to allele G./Genotype GG is associated with increased response to salvianolic acid b in people with Coronary Disease as compared to genotypes GT + TT./Genotypes GT + TT is associated with decreased expression of NOS3 mRNA as compared to genotype GG.	22890915
NPPA	rs5065	stoplost/ctcf	0.18	Chlorthalidone	Unknown	Allele G is associated with decreased major adverse cardiac events (mace) when treated with chlorthalidone in people with Hypertension as compared to genotype AA.	18212314
NR3C2	rs5522	missense/enhancer	0.11	Enalapril	Unknown	Genotype TT is associated with increased response to enalapril in people with Hypertension as compared to genotypes CC + CT.	24059494
NT5C2	rs10883841	missense	0.08	Peginterferon alfa-2a		Genotype CT is associated with decreased likelihood of cryoglobulinemia when treated with peginterferon alfa-2a in people with as compared to genotype TT.	28453396
P2RY12	rs6785930	missense	0.24	Clopidogrel	Completed	Allele A is associated with increased risk of neurological events when treated with clopidogrel in people with Peripheral Vascular Diseases as compared to genotype GG, etc.	15933261
P2RY12	rs6809699	synonymous	0.09	Clopidogrel	Completed	Genotypes AA + AC is associated with increased resistance to clopidogrel in people with Coronary Disease as compared to genotype CC.	PMC9801627
PCSK9	rs11591147	missense/promoter	0.01	Atorvastatin	Completed	Allele T is associated with LDL-C response when treated with atorvastatin in people with Coronary Disease.	20031582
PON1	rs662	missense	0.46	Simvastatin	Completed	Allele C is associated with increased risk of major adverse cardiac events (mace) when treated with clopidogrel as compared to allele T./Genotype CC is associated with increased response to simvastatin in people with Coronary Disease as compared to genotype TT, etc.	PMC4752331
PTGS1	rs3842787	missense/promoter	0.06	Rofecoxib	Unknown	Allele T is associated with reduction in COX-1 inhibition and depression of the urinary thromboxane metabolite when exposed to rofecoxib in healthy individuals as compared to allele C.	16401468
SCARB1	rs4238001	missense/promoter	0.06	Fenofibrate	Unknown	Genotypes CT + TT are associated with increased response to fenofibrate in people with Hypertriglyceridemia as compared to genotype CC.	PMC3836273
SCARB1	rs5888	stoplost	0.32	Atorvastatin	Completed	Genotype AA is associated with increased response to atorvastatin in people with Hypercholesterolemia as compared to genotypes AG + GG.	20064494
SH2B3	rs3184504	missense	0.15	Candesartan	Completed	Allele T is associated with increased response to candesartan in people with Hypertension as compared to allele C.	31327267
SLCO1B1	rs4149056	missense	0.09	Simvastatin	Completed	Allele C is associated with increased likelihood of Muscular Diseases when treated with simvastatin in people with Coronary Artery Disease as compared to allele T, etc.	PMC5728073
SLCO1B1	rs11045819	missense	0.06	Fluvastatin	Completed	Genotype CC is associated with decreased LDL-C reduction when treated with fluvastatin in people with Hypercholesterolemia as compared to genotypes AA + AC.	18781850
SLCO1B1	rs34671512	missense	0.04	Rosuvastatin	Completed	Allele C is associated with decreased exposure to rosuvastatin in healthy individuals as compared to allele A.	30100615
SLCO1B1	rs2306283	missense	0.38	Atorvastatin	Completed	Genotype AA is associated with increased clinical benefit to atorvastatin in people with Coronary Artery Disease as compared to genotypes AG + GG.	35968761
SLCO1B1	SLCO1B1*5 (rs4149056)	missense	0.09	Lovastatin acid	NA	SLCO1B1 *5/*15 + *15/*15 are associated with increased exposure to lovastatin acid in healthy individuals as compared to SLCO1B1 *1/*1.	26020121
TLR4	rs4986790	missense	0.06	Pravastatin	Completed	Genotypes AG + GG are associated with decreased risk of cardiovascular events when treated with pravastatin in men with Coronary Artery Disease as compared to genotype AA.	12742999
TLR4	rs4986791	missense	0.04	Prednisolone	Unknwon	Allele T is associated with decreased clinical benefit to prednisolone in children with Thrombocytopenia as compared to allele C.	37638833

MAF refers to minor allele frequency and etc means there is/are additional allelic/genotypic association(s) which is not stated here. Additionally, NA means Not Applicable.

**Table 2:** Variant annotation assessment including the regulatory variants of pharmacogenes-associated with CAD.

Genes	Variant	Function	MAF	Drugs	Association
ABCA1	rs2487032	enhancer	0.4	clopidogrel	Allele A is associated with increased metabolism of clopidogrel as compared to genotype GG.
ABCB1	rs3213619	promoter	0.05	atenolol	Allele G is associated with increased risk of Hypercholesterolemia due to atenolol in people with Hypertension as compared to allele A.
ACE	rs4291	promoter	0.35	amlodipine; chlorthalidone; lisinopril	Genotypes AA + AT are associated with decreased fasting glucose when treated with amlodipine, chlorthalidone or lisinopril in people with Hypertension as compared to genotype TT.
ACE2	rs2106809	intronic/promoter	0.32	captopril	Genotype GG is associated with increased response to captopril in women with Hypertension as compared to genotypes AA + AG.
ADIPOQ	rs266729	TFB site	0.23	Antihypertensives	Genotypes CG + GG is associated with increased severity of Hypertension when treated with Antihypertensives in people with Hypertension as compared to genotype CC.
AGT	rs5051	promoter	0.29	atenolol	Genotypes CT + TT are associated with increased response to atenolol in people with Hypertension as compared to genotype CC, etc.
AGT	rs5050	promoter	0.18	aspirin	Genotype GG is associated with increased risk of Peptic Ulcer Hemorrhage when treated with aspirin as compared to genotypes GT + TT.
APOC1	rs4420638	enhancer	0.15	hmg coa reductase inhibitors	Allele G is associated with decreased response to hmg coa reductase inhibitors in people with Cardiovascular Diseases or Hypercholesterolemia as compared to allele A.
APOE	rs449647	ctcf	0.2	atorvastatin; bezafibrate	Genotypes AT + TT is associated with increased lipid-lowering effect when treated with atorvastatin or bezafibrate in people with Hyperlipidemias as compared to genotype AA.
APOE	rs71352238	promoter	0.09	rosuvastatin	Genotypes CC + CT are associated with decreased response to rosuvastatin as compared to genotype TT.
ATP2B1	rs12817819	enhancer	0.07	Antihypertensives	Genotypes CT + TT are associated with increased resistance to Antihypertensives in people with Coronary Artery Disease or Hypertension as compared to genotype CC.
CCND1	rs9344	splicing	0.41		Genotypes AA + AG is associated with increased transcription of CCND1 mRNA as compared to genotype GG.
CES1	rs8192935	splicing	0.42	dabigatran	Genotypes AA + AG is associated with decreased dose-adjusted trough concentrations of dabigatran in people with Atrial Fibrillation as compared to genotype GG.
CETP	rs708272	ctcf/enhancer	0.38	hmg coa reductase inhibitors	Allele A is associated with decreased risk of cardiovascular events when treated with hmg coa reductase inhibitors in people with Coronary Artery Disease as compared to allele G, etc.
CETP	rs4783961	enhancer	0.44	fluvastatin	Genotype GG is associated with increased response to fluvastatin in people with Hypercholesterolemia as compared to genotypes AA + AG.
CETP	rs3764261	enhancer	0.29	hmg coa reductase inhibitors	Allele A is associated with increased HDL cholesterol when treated with hmg coa reductase inhibitors.
F2R	rs168753	splice polypyrimidine tract variant	0.18	aspirin; clopidogrel	Allele T is associated with decreased platelet reactivity when treated with aspirin and clopidogrel in people with Ischemic Attack, Transient or Stroke as compared to allele A.
IL6	rs1800795	intronic/promoter	0.14	fenofibrate	Genotypes CC + CG are associated with increased response to fenofibrate in patients with a high risk of cardiovascular disease as compared to genotype GG.
KDR	rs7667298	promoter	0.5	clopidogrel	Genotype CC is associated with decreased likelihood of Angina Pectoris when treated with clopidogrel in people with Coronary Disease as compared to genotypes CT + TT.
KDR	rs2305948	promoter	0.5	clopidogrel	Allele T is associated with decreased response to clopidogrel in people with Coronary Disease as compared to allele C, etc.
KL	rs36217263	promoter	0.44	Beta Blocking Agents	Genotypes A/del + del/del are associated with decreased response to Beta Blocking Agents in people with Hypertension as compared to genotype AA.
LIPC	rs1800588	intronic/enhancer	0.39	simvastatin	Genotype CT is associated with increased response to simvastatin as compared to genotype TT, etc.
MED12L; P2RY12	rs10935838	intronic/enhancer	0.13	clopidogrel	Genotype AG is associated with decreased platelet aggregation when exposed to clopidogrel in healthy individuals as compared to genotype GG.
NFE2L2	rs6721961	intronic/promoter	0.15	estradiol	Allele T is associated with increased risk of venous thromboembolism when treated with estradiol as compared to genotype CC.
NOS3	rs2070744	intronic/ctcf	0.23	atorvastatin	Genotype CC is associated with decreased erythrocyte plasma membrane fluidity when treated with atorvastatin in healthy individuals.
NPC1L1	rs17655652	promoter	0.15	pravastatin	Genotype CC is associated with increased reduction in LDL-C when treated with pravastatin in men as compared to genotypes CT + TT.
PON1	rs705379	intronic/promoter	0.35	atorvastatin; simvastatin	Genotypes AA + AG are associated with change in HDL-cholesterol when treated with atorvastatin or simvastatin in people with Hypercholesterolemia.
PTGS1	rs10306114	promoter	0.05	aspirin	Allele G is associated with increased risk of non-response to aspirin when treated with aspirin in people with Coronary Artery Disease as compared to genotype AA, etc.
PTGS2	rs20417	promoter	0.2	aspirin	Allele G is associated with decreased risk of Coronary Disease when treated with aspirin as compared to allele C, etc.
SLCO1B1	rs2291073	intronic/enhancer	0.29	lovastatin	Genotypes GT + TT are associated with increased response to lovastatin as compared to genotype GG.
SREBF1	rs60282872	promoter	0.46	fluvastatin	Genotype CC is associated with increased apolipoprotein A1 and C3 when treated with fluvastatin in people with Coronary Artery Disease.
THBD	rs1042580	promoter	0.3	warfarin	Genotypes CC + CT is associated with decreased risk of Hemorrhage when treated with warfarin as compared to genotype TT.
TNF	rs1800629	promoter	0.09	atorvastatin	Genotypes AA + AG is associated with increased activity of TNF as compared to genotype GG./Genotype GG is associated with increased bone marrow density in the lumbar spine when treated with atorvastatin in people with Acute coronary syndrome.

TFB means transcription factor binding; MAF refers to minor allele frequency and etc means there is/are additional allelic/genotypic association(s) which is not stated here.

**Table 3:** Variant annotation assessment including non-coding variants of pharmacogenes-associated with CAD.

Genes	Variant	Function	MAF	Drugs	Association
ABCA1	rs12003906	intronic	0.07	atorvastatin; pravastatin; simvastatin	Allele C is associated with decreased response to atorvastatin, pravastatin or simvastatin in people with Hyperlipidemias as compared to allele G.
ABCA1	rs2472507	intronic	0.23	–	Allele C is associated with increased expression of ABCA1 in HapMap cells.
ABCA1	rs2515629	intronic	0.14	–	Allele G is associated with increased expression of ABCA1 in HapMap cells.
ABCA1	rs4149297	intronic	0.08	–	Allele G is associated with increased expression of ABCA1 in HapMap cells.
ABCB1	rs10267099	intronic	0.14	atenolol	Allele G is associated with increased risk of Hypercholesterolemia due to atenolol in people with Hypertension as compared to allele A.
ABCB1	rs1922242	intronic	0.38	fluvastatin	Genotype AA is associated with increased response to fluvastatin in people with Hypercholesterolemia as compared to genotype AT.
ABCG1	rs225440	intronic	0.43	fluorouracil; irinotecan; leucovorin	Allele T is associated with increased risk of Neutropenia when treated with fluorouracil, irinotecan and leucovorin in people with Colorectal Neoplasms as compared to genotype CC.
ACE	rs4341	3utr	0.47	pravastatin	Genotypes CG + GG are associated with increased response to pravastatin as compared to genotype CC.
ACE	rs1799752	intronic	na	quinapril	Genotypes ATACAGTCACTTTTTTTTTTTTTTTGAGACGGAGTCTCGCTCTGTCGCCC/del + del/del are associated with increased response to quinapril in people with Coronary Artery Disease as compared to genotype ATACAGTCACTTTTTTTTTTTTTTTGAGACGGAGTCTCGCTCTGTCGCCC/ATACAGTCACTTTTTTTTTTTTTTTGAGACGGAGTCTCGCTCTGTCGCCC.
ACE	rs4344	intronic	0.47	ramipril	Genotypes AA + GG are associated with increased response to ramipril in people with Hypertension as compared to genotype AG.
ACE	rs4359	intronic	0.42	ramipril	Genotypes CC + TT are associated with increased response to ramipril in people with Hypertension as compared to genotype CT.
AGT	rs7079	3utr	0.19	benazepril	Genotype TT is associated with increased response to benazepril in people with Hypertension as compared to genotypes GG + GT, etc.
AGT	rs943580	intronic	0.28	Antiinflammatory agents, non-steroids	Genotype GG is associated with decreased likelihood of Acute coronary syndrome when exposed to Antiinflammatory agents, non-steroids in people with Acute coronary syndrome as compared to genotypes AA + AG.
APOA1	rs964184	3utr	0.22	fenofibrate	Allele G is associated with increased response to fenofibrate in people with Hypertriglyceridemia as compared to allele C.
APOA1	rs2727786	intronic	na	fenofibrate	Genotype CG is associated with decreased response to fenofibrate in people with Hypertriglyceridemia as compared to genotype CC.
APOA5	rs662799	intergenic	0.16	atorvastatin; rosuvastatin; simvastatin	Genotype AA is associated with response to atorvastatin, rosuvastatin and simvastatin in people with Dyslipidaemia as compared to genotypes AG + GG.
APOB	rs679899	3utr	0.48	warfarin	Allele G is associated with increased risk of Hemorrhage when treated with warfarin in people with heart valve replacement as compared to allele A.
APOC1; APOE	rs445925	ncRNA	0.15	–	Allele A is associated with baseline LDL cholesterol in people with Vascular Diseases.
APOC3	rs5128	3utr	0.23	protease inhibitors	Genotypes CG + GG are associated with decreased Hypertriglyceridemia when treated with protease inhibitors as compared to genotype CC.
APOC3	rs2854116	intergenic	0.45	protease inhibitors	Genotypes CC + CT are associated with decreased severity of Hypertriglyceridemia when treated with protease inhibitors as compared to genotype TT.
APOC3	rs2854117	intergenic	0.5	protease inhibitors	Genotypes CT + TT are associated with decreased severity of Hypertriglyceridemia when treated with protease inhibitors as compared to genotype CC.
ATP2B1	rs17381194	intronic	0.06	hmg coa reductase inhibitors	Allele T is associated with increased risk of Myalgia unspecified when treated with hmg coa reductase inhibitors in people with Hyperlipidemias as compared to allele C.
CD80	rs34394661	intergenic	0.23	clopidogrel	Genotype AA is associated with increased likelihood of high on-treatment platelet reactivity when treated with clopidogrel in people with Acute coronary syndrome as compared to genotypes AG + GG.
CDH13	rs11859453	intronic	0.43	aspirin; clopidogrel	Genotypes AA + AG is associated with decreased likelihood of Coronary Restenosis or Disease Progression when treated with aspirin and clopidogrel in people with Coronary Disease as compared to genotype GG.
CELSR2	rs646776	intergenic	0.24	hmg coa reductase inhibitors	Allele C is associated with increased response to hmg coa reductase inhibitors as compared to allele T.
CELSR2; PSRC1	rs602633	intergenic	0.25	–	Allele T is associated with baseline LDL cholesterol in people with Vascular Diseases.
CES1	rs8192950	intronic	0.37	clopidogrel	Allele G is associated with decreased risk of Ischemic Attack, Transient and Stroke when treated with clopidogrel in people with Constriction, Pathologic as compared to allele T.
CES1	rs2244613	intronic	0.33	dabigatran	Genotypes GG + GT are associated with decreased risk of bleeding events when treated with Dabigatran in people with Atrial Fibrillation as compared to genotype TT, etc.
CETP	rs1532624	intron	0.31	hmg coa reductase inhibitors	Genotype AA is associated with decreased response to hmg coa reductase inhibitors in people with Hyperlipidemias as compared to genotype CC.
CRP	rs1205	3utr	0.34	rosuvastatin	Genotypes CT + TT are associated with increased likelihood of Acute coronary syndrome when exposed to Antiinflammatory agents, non-steroids in people with Acute coronary syndrome as compared to genotype CC, etc.
CYP1A2	rs762551	intronic	0.37	clopidogrel	Genotypes AA + AC are associated with decreased on-treatment platelet reactivity when treated with clopidogrel in people with cigarette smokers as compared to genotype CC.
CYP2B6	rs70950385	3utr	na	–	Genotype CA/CA is associated with decreased activity of CYP2B6 as compared to genotype AG/AG.
CYP7A1	rs3808607	intergenic	0.48	atorvastatin	Genotype TT is associated with increased mean percentage reduction of triglycerides level when treated with atorvastatin in people with Hyperlipidemias as compared to genotypes GG + GT.
F3	rs841698	intronic	0.09		Allele T is associated with increased expression of ABCD3 in HapMap cells.
F3	rs3917643	intronic	0.02	simvastatin	Genotype CT is associated with increased response to simvastatin in people with Myocardial Ischemia as compared to genotype TT.
F7	rs510317	intergenic	0.23	warfarin	Genotypes AA + AG are associated with increased dose of warfarin as compared to genotype GG.
F7	rs510335	intergenic	0.2	warfarin	Genotypes GT + TT are associated with decreased warfarin dose when treated with warfarin as compared to genotype GG.
FADS1; FEN1; MIR611; TMEM258	rs174541	ncRNA	0.3	–	Allele T is associated with baseline LDL cholesterol in people with Vascular Diseases.
GALNT2	rs2144300	intronic	0.33	atenolol	Allele C is associated with Hypercholesterolemia due to atenolol in people with Hypertension as compared to allele T.
GALNT2	rs2144297	intronic	0.43	atenolol	Allele T is associated with Hypercholesterolemia due to atenolol in people with Hypertension as compared to allele C.
HMGCR	rs17671591	intergenic	0.4	atorvastatin	Allele T is associated with LDL-C response when treated with atorvastatin in people with Coronary Disease./Genotypes CT + TT is associated with increased response to atorvastatin in people with Hypercholesterolemia as compared to genotype CC.
HMGCR	rs12654264	intronic	0.44	–	Genotypes AT + TT are associated with increased serum total cholesterol as compared to genotype AA.
HMGCR	rs10474433	intronic	0.4	atorvastatin	Allele T is associated with LDL-C response when treated with atorvastatin in people with Coronary Disease.
HMGCR	rs17244841	intronic	0.04	pravastatin	Genotype AT is associated with decreased reduction in total cholesterol when treated with pravastatin as compared to genotype AA./Genotype AA is associated with increased reduction of LDL cholesterol when treated with simvastatin.
HMGCR	rs3846662	ncRNA	0.38	–	Genotype GG is associated with increased low-density lipoprotein cholesterol level in basal state and possibly in response to atorvastatin. when treated with atorvastatin in healthy individuals as compared to genotype AA, etc.
HMGCR	rs17238540	ncRNA	0.04	pravastatin	Genotype GT is associated with decreased reduction in total cholesterol when treated with pravastatin as compared to genotype TT.
IL1B	rs16944	intergenic	0.49	pravastatin	Genotype GG is associated with increased coronary flow reserve when treated with pravastatin in men as compared to genotypes AA + AG.
KL	rs211247	intergenic	0.15	Antiinflammatory agents, non-steroids	Genotypes CG + GG are associated with increased likelihood of Acute coronary syndrome when exposed to Antiinflammatory agents, non-steroids in people with Acute coronary syndrome as compared to genotype CC.
LPA	rs10455872	intronic	0.02	hmg coa reductase inhibitors	Genotypes AG + GG are associated with increased risk of Coronary Artery Disease when treated with hmg coa reductase inhibitors as compared to genotype AA./Allele G is associated with increased risk of Coronary Disease when treated with hmg coa reductase inhibitors as compared to allele A, etc.
MED12L; P2RY12	rs5853517	intronic	0.24	clopidogrel	Genotype T/del is associated with decreased platelet aggregation when exposed to clopidogrel in healthy individuals as compared to genotype del/del.
MED12L; P2RY12	rs6801273	intronic	0.42	clopidogrel	Allele T is associated with decreased residual on-clopidogrel platelet reactivity when treated with clopidogrel in people with Coronary Artery Disease as compared to allele C.
MME	rs989692	5utr	0.39	Ace Inhibitors, Plain	Genotypes CT + TT is associated with increased likelihood of Angioedema when treated with Ace Inhibitors, Plain as compared to genotype CC.
MME	rs2016848	intronic	0.45	Ace Inhibitors, Plain	Allele A is associated with increased risk of Cough when treated with Ace Inhibitors, Plain in people with Hypertension as compared to allele G.
MMP3	rs35068180	intergenic	0.49	pravastatin	Genotypes A/del + AA are associated with increased response to pravastatin in men with Coronary Artery Disease.
MTTP	rs1800591	intronic	0.25	atorvastatin	Genotype TT is associated with increased response to atorvastatin in men with Hyperlipoproteinemia Type II.
NPC1L1	rs2072183	ncRNA	0.25	hmg coa reductase inhibitors	Allele G is associated with decreased response to hmg coa reductase inhibitors in people with Cardiovascular Diseases and Hypercholesterolemia as compared to allele C.
NT5C2	rs11191612	intronic	0.34	–	Genotype GG is associated with increased transcription of NT5C2 as compared to genotypes AA + AG.
P2RY12	rs2046934	intronic	0.13	–	Allele G is associated with increased risk of Coronary Artery Disease and platelet aggregation as compared to allele A, etc.
P2RY12	rs9859552	intronic	0.06	cangrelor	Genotype TT is associated with 20% and 17% less inhibition of platelet aggregation with crangrelor (0.05 and 0.25 microM) in-vitro when exposed to cangrelor as compared to genotype GG.
P2RY12	rs6787801	intronic	0.47	clopidogrel	Allele G is associated with decreased residual on-clopidogrel platelet reactivity when treated with clopidogrel in people with Coronary Artery Disease as compared to allele A.
P2RY12	rs3732759	intronic	0.32	clopidogrel	Genotype GG is associated with decreased response to clopidogrel in people with Coronary Disease as compared to genotypes AA + AG.
PTGS1	rs10306135	5utr	0.12	Antiinflammatory agents, non-steroids	Genotype TT is associated with increased likelihood of Acute coronary syndrome when exposed to Antiinflammatory agents, non-steroids in people with Acute coronary syndrome as compared to genotypes AA + AT.
PTGS1	rs1330344	intergenic	0.37	clopidogrel	Allele T is associated with increased risk of major adverse cardiac events (mace) when treated with clopidogrel as compared to allele C.
PTGS1	rs12353214	intergenic	0.15	Antiinflammatory agents, non-steroids	Genotype TT is associated with increased likelihood of Acute coronary syndrome when exposed to Antiinflammatory agents, non-steroids in people with Acute coronary syndrome as compared to genotypes CC + CT.
PTGS2	rs4648287	intronic	0.05	atenolol	Allele G is associated with increased likelihood of Hypercholesterolemia due to atenolol in people with Hypertension as compared to allele A.
PTGS2	rs4648276	ncRNA	0.11	Coxibs	Genotypes AG + GG are associated with increased likelihood of Acute coronary syndrome when exposed to Coxibs in people with Acute coronary syndrome as compared to genotype AA.
REN	rs11240688	intronic	0.24	hydrochlorothiazide	Genotype GG is associated with increased reduction in SBP when treated with hydrochlorothiazide in people with Essential hypertension as compared to genotypes AA + AG.
RHOA	rs11716445	intronic	0.03	pravastatin; simvastatin	Allele A is associated with decreased response to pravastatin or simvastatin in people with Hypercholesterolemia as compared to allele G.
SLCO1B1	rs113681054	intergenic	0.22	ticagrelor	Allele C is associated with increased concentrations of ticagrelor in people with Acute coronary syndrome as compared to allele T.
SLCO1B1	rs4149015	intronic	0.05	pravastatin	Genotype AG is associated with decreased response to pravastatin as compared to genotype GG.
SLCO1B1	rs58310495	intronic	0.17	fluvastatin	Allele T is associated with increased concentrations of fluvastatin in healthy individuals as compared to allele C.
SLCO1B1	rs4363657	intronic	0.21	hmg coa reductase inhibitors	Genotypes CC + CT are associated with increased likelihood of statin-related myopathy when treated with hmg coa reductase inhibitors in people with Cardiovascular Diseases as compared to genotype TT.
SLCO1B1	rs11045874	intronic	0.21	rosuvastatin	Allele C is associated with decreased exposure to rosuvastatin in healthy individuals as compared to allele G.
SLCO1B1	rs4149036	intronic	0.35	atorvastatin	Genotypes AC + CC are associated with increased response to atorvastatin as compared to genotype AA.
SLCO1B1	rs4149081	intronic	0.22	simvastatin	Genotype AA is associated with increased LDL-C reduction when treated with simvastatin in people with Coronary Disease as compared to genotypes AG + GG, etc.
SLCO1B1	rs2900478	intronic	0.09	hmg coa reductase inhibitors	Allele A is associated with decreased response to hmg coa reductase inhibitors as compared to allele T.
SLCO1B1	rs11045854	ncRNA	0.02	rosuvastatin	Allele A is associated with decreased exposure to rosuvastatin in healthy individuals as compared to allele G.
SORT1	rs629301	3utr	0.24	hmg coa reductase inhibitors	Allele T is associated with decreased response to hmg coa reductase inhibitors in people with Cardiovascular Diseases and Hypercholesterolemia as compared to allele G.
VASP	rs10995	3utr	0.22	hydrochlorothiazide	Genotypes AG + GG are associated with increased response to hydrochlorothiazide in people with Hypertension as compared to genotype AA.
VEGFC	rs1002976	intergenic	0.42	uric acid	Allele C is associated with increased concentrations of uric acid in people with Hypertension as compared to allele T.

MAF refers to minor allele frequency and etc means there is/are additional allelic/genotypic association(s) which is not stated here.

## 4 Discussion

In industrialized nations, the occurrence of CAD continues to decrease, but owing to population migration and aging, the overall rate of coronary events and, as a result, the incidence of CAD will not decrease, but might even rise in the near future. The prevalence of CAD varies greatly among developing nations. The globalization of the Western diet and increasing sedentary lifestyle will have a profound influence on these nations' gradual growth in the prevalence of CAD. The gradual decline in CAD mortality in industrialized nations over the last few decades may be attributed to both efficient acute-phase therapies and enhanced primary and secondary prevention strategies [7]. Meanwhile, ethnical differences, social inequities, and disparities in the availability of efficient therapies and preventative measures in various regions of the same nation might all have an impact on overall outcomes, necessitating greater investigation and assessment of different locations inside the country [36]. As mentioned before, the findings of single and GWAS studies on CAD are increasingly utilized in personalized medicine and pharmacogenomics strategies of CAD treatment. Thus, this review systematically studied the recent reports from 2020 to 2023 first and then performed a remarkable in silico analysis on a large amount of data. In summary, 5618 articles were found, 1290 papers were eligible, 650 papers were included, 4608 protein-coding genes were extracted, 1432 unique genes distinguished, 530 evidence-based repeated genes remained, among them 71 genes had a pharmacogenetics variant at least (totally 6331 annotations), VAA indicated that 532 variants had potentials to be considered for final report, and lastly, final PGs list according to the deeply matched annotations with CAD revealed 175 variants. According to the function and MAF, 57 structural variants (missense) of 29 PGs remained associated with CAD.

Numerous studies reported the genetic correlations of CAD in both small-sized (single or multiple genes) and GWAS results. Veluchamy et al. (2019) indicated, for example, that among two new genome-wide significant loci relating to the COL4A2 gene for retinal arteriolar tortuosity and gene for retinal venular tortuosity. ACTN4/CAPN12 has been linked to CAD and the pathophysiology of atrial fibrillation [37]. Zhao et al. (2018) discovered a significant relationship between variants situated on cytogenetic position 15q26.1 and CAD risk. The potential CAD-related SNP at this locus is found in the FURIN gene's non-coding region. FURIN is primarily produced by macrophages and also is expressed in atherosclerotic plaques, according to a previous study [38]. In another study, Li et al. (2018) discovered three new intragenic SNP that were associated with CAD. Only two of these had transcriptional impacts and were linked with lower expression levels of the SCML4 and THSD7A genes, which are risk alleles and protective alleles, respectively. Employing short interfering RNA to limit translation, they found that knocking down SCML4 boosted the IL-6 levels, E-selectin, and also ICAM, making endothelial cells more susceptible to apoptosis. Additionally, adenovirus-mediated short hairpin RNA suppression of SCML4 resulted in considerably decreased luminal section area compared to controls in a rat study involving partial carotid ligation. Independently, they found that knocking down THSD7A using a short interfering RNA reduced monocyte adhesion by lowering the expression level of ICAM, ITGB2, L-selectin, and in a monocyte/macrophage line [39].

Notably, the current study reviewed the PubMed publications on the topic of “coronary artery disease pharmacogenomics” which showed 29 papers from 2001 to 2023, and the relevant and important reports are summarized here. Kajinami et al. (2005) conducted a review of the Pharmacogenomics of Statin Responsiveness and identified genetic variation at gene loci affecting intestinal cholesterol absorption, including apolipoprotein (APO) E; adenosine triphosphate-binding cassette transporter G5 and G8; cholesterol production, like as 3-hydroxy-3-methylglutaryl coenzyme A reductase; and lipoprotein catabolism, such However, there is a significant variation in the findings reported, and the data recommended that merged analysis of multiple genetic variants in several genes, all of which have potential functional relevance, is more probable to yield significant findings than single gene studies in small sample groups [40]. Tsikouris and Peeters (2007) reviewed the existing Renin-Angiotensin System (RAS) inhibitor pharmacogenomic investigations that have assessed RAS variants that either reveal mechanisms through surrogate outcome measures or predict effectiveness through clinical results in CAD-related disorders. Despite the outcome, none of the RAS genotypes predicts RAS inhibitor effectiveness decisively [41]. Inter-individual variability in pharmacokinetics and pharmacodynamics has been demonstrated to alter the clinical result of long-term coronary artery disease therapy, according to Remmler and Cascorbi (2008). They highlighted the effects of Plasminogen activator inhibitor type I (PAI-I), Fibrinogen, ACE, eNOS, APOB, APOE, APO-AV, APO-CIII, Lipoprotein lipase and hepatic lipase (LIPC), OLR1, CETP, OATP1B1, CYP2C9, ADRB1, ADRB2, ADR2AC, and VKORC1 [42]. According to Ellis et al. (2009), clopidogrel antiplatelet treatment is the standard care for CAD patients having percutaneous coronary intervention. Yet, about 25% of individuals have a subtherapeutic antiplatelet response. Clopidogrel is a prodrug which is biotransformed into its active metabolite by CYP2C19 in the liver. Numerous investigations have found that, when compared to wild-type individuals, CYP2C19 variant allele carriers have a considerably reduced capacity to convert clopidogrel to its active metabolite and reduce platelet activation, putting them at a significantly greater risk of adverse cardiovascular events [43]. Homeyer and Schwinn (2011) investigated the Pharmacogenomics of -Adrenergic Receptor Physiology and Response to -Blockade and found two clinically significant SNPs for the 1AR (Ser49Gly, Arg389Gly), three for the β2AR (Arg16Gly, Gln27Glu, Thr164Ile), and one for the β3AR (Trp64Arg). They stated that, whereas AR SNPs may not directly cause disease, they do appear to be risk factors for the condition as well as regulators of disease and response to stress and medications. This has been observed especially in the perioperative context for the Arg389Gly β1AR polymorphism, with individuals with the Gly variation having a greater frequency of unfavorable perioperative outcomes [44]. Luchessi et al. (2013) discovered that the difference in response to Acetylsalicylic acid (ASA) may be associated with an elevated level of IGF1 and IGF1R, as well as a response to clopidogrel can be influenced by pharmacokinetic changes corresponding to the reverse transport pathway via raised expression of ABCC3 [45]. The most consistent findings in a study by Yasmina et al. (2014) included clopidogrel, where CYP2C19 loss-of-function alleles were connected to stent thrombosis occurrences. In patients with CAD having percutaneous coronary intervention and stenting, they advise genotyping for CYP2C19 loss-of-function alleles and modifying the antiplatelet regimen depending on the genotyping outcomes [46]. Ssaydam et al. (2017) aimed to identify the most notable mutations in the genes implicated in the pharmacokinetics and pharmacodynamics of clopidogrel. Their study comprised 347 Turkish patients who underwent percutaneous coronary procedures with stent insertion. Genotyping was performed on variations in the CYP2C19, CYP3A4, CYP2B6, ABCB1, ITGB3, and PON1 genes. The CYP2C19*2 (G636A) polymorphism was shown to be associated with non-responsiveness to clopidogrel medication (p < 0.001). In non-responders, the allele frequency of this SNP was substantial; its odds ratio was 2.92 compared to the G allele (p < 0.001). Their results revealed that the CYP2C19*2 polymorphism is related to non-responsiveness to clopidogrel treatment, whereas the CYP2C19*17 polymorphism increases clopidogrel's antiplatelet action. Clopidogrel-treated individuals can be protected or not against stent thrombosis and ischemic events based on the haplotypes of these two SNPs [47]. Fragoulakis et al. (2019) compared pharmacogenomics-guided clopidogrel medication to non-pharmacogenomics-guided clopidogrel treatment for coronary artery syndrome subjects having percutaneous coronary intervention (PCI) in the Spanish healthcare context. A total of 549 individuals with CAD who had PCI were selected. Their findings showed that a pharmacogenomics-guided clopidogrel therapy might be a more cost-effective option for patients undergoing PCI than a non-pharmacogenomics-guided procedure [48]. Verma et al. (2020) used samples from 2750 European people to conduct a GWAS. GWAS for platelet reactivity indicated a significant signal for CYP2C19*2 (P value = 1.67e33), and mutations in SCOS5P1, CDC42BPA, and CTRAC1 proved genome-wide significance in the CAD, percutaneous coronary intervention, and acute coronary syndrome subgroups (lowest P values: 1.07e-09, 4.53e-08, and 2.60e-10, respectively). They concluded that CYP2C19*2 is the most powerful genetic factor of on-clopidogrel platelet responsiveness [49]. In their narrative review (2021), Hirata et al. examined pharmacogenomic research on antithrombotic medications routinely administered in Brazil. Few pharmacogenomics studies have looked at antiplatelet drugs in Brazilian cohorts, and they discovered relationships between CYP2C19*2, PON1 rs662, and ABCC3 rs757421 genotypes and platelet responsiveness or clopidogrel pharmacokinetic (PK) in participants suffered from CAD or acute coronary syndrome (ACS), whereas ITGB3 contributes to aspirin PK but not platelet responsiveness in diabetic individuals. Brazilian anticoagulant and antiplatelet guidelines recommended using a platelet aggregation evaluation or genotyping only in determined cases of ACS individuals taking clopidogrel who do not have ST-segment elevation, and they additionally suggested CYP2C9 and VKORC1 genotyping before establishing warfarin therapy to evaluate the risk of bleeding incidents or warfarin resistance [50].

Statin medications have been utilized for years in the main and second-line prevention of CAD due to their ability to decrease cholesterol. According to recent studies, the positive benefits of statins expand over their lipid-lowering impacts and may potentially protect against atherosclerosis through lipid-lowering unrelated pathways [51,52]. Yu and his colleagues demonstrated that rosuvastatin treatment reduced coronary artery atherosclerosis, platelet accumulation in atherosclerotic coronary arteries, cardiomegaly, and cardiac fibrosis in a mouse model that spontaneously had coronary artery atherosclerosis. Regardless of rising plasma cholesterol levels, these beneficial effects included a reduction in accumulated oxidized phospholipids in damaged artery walls as well as a reduction in macrophage foaming production [53]. The endothelium has a remarkable impact on healing wounds and serves such a barrier to regulate the transportation of leukocytes. The endothelium is essential for wound healing and works as a barrier to limit leukocyte transportation [54,55]. The modification of endothelial function, which is critical to wound healing and as a barrier in the atherosclerotic progression, is a key role of statin medicines. In atherosclerosis before MI, endothelial barrier function is compromised. After MI, Leenders et al. looked at how statins affected the function of the endothelium barrier in atherosclerotic ApoE-deficient mice. Statin treatment reduced infarcted tissue porosity and the entry of undesirable inflammatory leukocytes. On day 21 after an MI, hearts treated with statins performed better as a result of this. After MI, statin treatment improved the infarct endothelial barrier's performance and prevented the progression of scarring. This study demonstrated the importance of statin therapy for infarct repair after MI [56].

Human genetics has been extensively employed in recent years to determine causal relationships of hypothesized biomarkers or drug targets. Mendelian randomization (MR) studies utilize genetic variations to assess the causation of a disease's hypothesized risk variables [57]. If genetic variations have no further instant impact other than modulating a risk factor (for example LDL cholesterol) and also show a connection with downstream characteristics (for instance CAD), that second condition is most likely initiated with the risk factor (LDL-C), demonstrating a causal link between the risk factor and CAD [[58], [59], [60], [61], [62]]. As a result of its independence from confusion, MR research goes beyond standard epidemiological studies in determining causality [63]. Several conventional CAD risk factors, including triglycerides, LDL-C, LP(a), obesity, blood pressure, alcohol consumption, smoking, and T2D, are strengthened by MR. MR validated the presence of multiple causative risk factors, particularly APOC3, IL-1, IL6, insulin resistance, height, non-fasting glucose, telomere length, HMGCR, and Niemann-Pick C1-Like 1 (NPC1L1) [23]. Additionally, MR can assess the impact of drugs in a tailored setting. As an instance, Ference et al. used MR to analyze the impact of LDL-C reduction on the CAD risk caused by variants in NPC1L1 (ezetimibe target), HMGCR (statins target), or both (combinational treatment with ezetimibe and a statin) [64]. Furthermore, MR studies may expedite the lengthy process of obtaining FDA approval for a drug. MR investigations can reveal if drug targets are causal and how they are modulated with side effects. MR investigations are affordable, quick, and straightforward to conduct. Indeed, MR studies may be more precise than randomized controlled trials and are thus advised for therapeutic targets before moving to clinical trials [23].

Interestingly, PPI and GMI (Fig. 3, Fig. 4) showed a common miRNA, hsa-miR-33a. Interestingly, Reddy et al., in 2019 demonstrated a significant relationship between amplified amounts of plasma miR-33 and CAD and concluded that plasma miR-33 seems to have a noticeable non-invasive biomarker role [65]. The findings of the current in silico analysis revealed strong associations of hsa-miR-33a with ABCA1 gene variants (Table 1, Table 2, Table 3) in all three categories including missense variants (rs2230806, rs2230808), regulatory variant (rs2487032 as enhancer), and non-coding variants (rs12003906, rs2472507, rs2515629, and rs4149297). Genotype TT of rs2230806 is associated with increased HDL-cholesterol when treated with pravastatin in people with CAD as compared to genotype CC. Genotype CC of rs2230808 is associated with increased response to fenofibrate in people with Hypertriglyceridemia as compared to genotype TT. Genotype AA and AG of rs2487032 is associated with increased metabolism of clopidogrel as compared to genotype GG. Allele C of rs12003906 is associated with decreased response to atorvastatin, pravastatin, or simvastatin in people with Hyperlipidemias as compared to allele G. Allele C, G, and G of rs2472507, rs2515629, and rs4149297, respectively are associated with increased expression of ABCA1 in HapMap cells. Moreover, Dong et al.‘s study confirmed the increased expression levels of miR-24, miR-33a, miR-103a, and miR-122 in peripheral blood mononuclear cells (PBMCs) with the incidence of CAD [66]. Among these miRNAs, hsa-miR-101 was in the center of the concentric GMI model represented in Fig. 3 which displayed 4 connections with *PTGS2, CCND1, RHOA*, and *VEGFC*.

As an important role of the mentioned drugs in the current study, the clinical trial status of these drugs with CAD is highly remarkable for drug prescribing based on patients’ genotypes. *ABCA1* (rs2230806) with Pravastatin, *ABCB1* (rs2032582 and rs1045642) and (rs1128503) with Simvastatin, *ALDH2* (rs671) with Nitroglycerin, *APOC1* (rs429358 and rs7412) with Warfarin, *APOE* (rs429358 and rs7412) with Warfarin, *CES1* (rs146456965, rs202001817, rs71647871, rs201065375, rs2307240, rs143718310, rs200707504, and rs151291296) with Clopidogrel, *CETP* (rs5882) with Simvastatin, *F7* (rs6046) with Warfarin, *FMO3* (rs1736557) with Clopidogrel, *ITGB3* (rs5918) with Aspirin, *LEPR* (rs1805094) with Atorvastatin and (rs1137101) with Simvastatin, *LPA* (rs3798220) with Aspirin, *P2RY12* (rs6785930 and rs6809699) with Clopidogrel, *PCSK9* (rs11591147) with Atorvastatin, *PON1* (rs662) with Simvastatin, *SCARB1* (rs4238001) with Fenofibrate and rs5888 with Atorvastatin, *SH2B3* (rs3184504) with Candesartan, *SLCO1B1* (rs4149056) with Simvastatin and (rs11045819) with Fluvastatin and (rs34671512) with Rosuvastatin and (rs2306283) with Atorvastatin, *TLR4* (rs4986790) with Pravastatin, *EDN1* (rs5370) with Atenolol, and *NOS3* (rs1799983) with Aspirin and Clopidogrel. These drugs are highly recommended for personalized medicine-based prescribing according to the genotyping of the 36 aforementioned variants. Compared to these genes, 11 genes have unknown clinical trials including *ABCG8, AGT, APOB, CYP2B6, CYP2C9, FABP1, KDR, NPPA, NR3C2, NT5C2*, and *PTGS1* which are highly suggested for future clinical trials of investigating the related drugs with CAD. Some of these genes had limited studies with pharmacology of CAD including *ALDH2* [67], *APOC1* ([68]), *FMO3* [69], *LEPR* [70], *P2RY12* [71], *SCARB1* [72], *SH2B3* [73], *SLCO1B1* [74], *TLR4* [75], *EDN1* [76], *NOS3* [77], *ABCG8* [78], *AGT* [79], *FABP1* [80], *KDR* [81], *NPPA* [82], *NR3C2* [83], *NT5C2* [84], and *PTGS1* [85].

## 5 Conclusion

In conclusion, GWAS and post-GWAS research on CAD has made significant advances toward comprehending the genetic framework of this complicated disease. In addition to identifying potential genes, these investigations aided drug development and enhanced disease risk assessment, in addition to preventative treatments. GWAS might lay the basis for CAD personalized medicine. Surprisingly, applications of post-GWAS will be strengthened by the incorporation of more OMIC data, plus personal and environmental impacts to provide a comprehensive understanding of this complicated disease, perhaps leading to the discovery of the missing heritability. To combine and comprehend such vast amounts of data, artificial intelligence, and deep learning algorithms will be essential. Based on the PPI, GMI, PDI, and VVA results and Reddy et al.‘s study, by evaluation of circulating miR33a in the plasma of individuals with CAD, and genotyping of pharmacogenomiclly actionable variants of ABCA1 gene including rs2230806, rs2230808, rs2487032, rs12003906, rs2472507, rs2515629, and rs4149297, precise prescriptions of CAD well-know drugs will be applicable. Altogether, the findings of this report can improve the importance of pharmacogenomics utilization in CAD for personalized treatment and candidate gene panel of PGx-CAD in WES and WGS analysis.

## Informed consent statement

Not applicable.

## Data availability

There is no data availability to state.

## CRediT authorship contribution statement

**Siamak Kazemi Asl:** Writing – review & editing, Supervision, Project administration, Conceptualization. **Milad Rahimzadegan:** Writing – original draft, Methodology, Investigation, Formal analysis, Data curation. **Alireza Kazemi Asl:** Visualization, Validation, Resources.

## Declaration of competing interest

The authors declare that they have no known competing financial interests or personal relationships that could have appeared to influence the work reported in this paper.
